# Mechanistic investigation of flavonoid-mediated inhibition of *N*-methyl-*N*-nitrosourea-induced mutagenicity via noncovalent DNA binding

**DOI:** 10.1186/s41021-026-00361-3

**Published:** 2026-06-10

**Authors:** Chisato Matsuoka, Nao Takatama, Takuya Muraoka, Keiko Inami

**Affiliations:** Division of Pharmaceutical Organic Chemistry, Faculty of Pharmaceutical Sciences, Sanyo-Onoda City University, Daigaku-dori 1-1-1, Sanyo-Onoda, Yamaguchi 756-0884 Japan

**Keywords:** Antimutagenicity, Isoliquiritigenin, Licoricidin, *N*^3^-methyladenine, *O*^6^-methylguanine, *N*-methyl-*N*-nitrosourea, Minor-groove binder, Intercalator, Sugar-backbone interaction

## Abstract

**Background:**

Previous research using the Ames assay showed that licoricidin (LCD) and isoliquiritigenin (ILTG) can block mutations caused by *N*-methyl-*N*-nitrosourea (MNU) in *Salmonella typhimurium* (*S. typhimurium*) TA1535. With the aim to clarify the antimutagenic mechanism of these flavonoids, we evaluated the noncovalent interactions of test compounds (LCD, ILTG, ethidium bromide (EtBr), or Hoechst 33258) with calf thymus DNA (ctDNA) using a UV-Vis spectrophotometer and quantified the DNA adducts formed when treated with a mixture of MNU and the test compounds. Additionally, we measured the half-life of MNU and the amount of each test compound remaining in the reaction mixture.

**Results:**

The spectral and thermodynamic parameters indicated considerable binding between flavonoids (LCD and ILTG) and DNA. Intercalative EtBr and the minor-groove binder Hoechst 33258 inhibited MNU-induced mutagenicity in *S. typhimurium* TA1535. *O*^6^-Methylguanine (*O*^6^-MeG) and *N*^3^-methyladenine (*N*^3^-MeA) were quantified in a mixture of ctDNA and the test compounds (LCD, ILTG, EtBr, and Hoechst 33258) using LC‒MS/MS. The amount of the DNA adducts decreased with increasing concentrations of the compounds. Moreover, the half-lives of MNU were similar in the presence and absence of flavonoids (LCD and ILTG). No new products were detected, and no significant changes in the flavonoids remaining in the reaction mixture were observed, indicating that LCD and ILTG did not react directly with MNU.

**Conclusions:**

LCD and ILTG directly bind to DNA and suppress DNA adduct formation. However, interaction with DNA alone does not fully account for their antimutagenic activity, implying the involvement of other mechanisms.

**Supplementary information:**

The online version contains supplementary material available at 10.1186/s41021-026-00361-3.

## Introduction

*N*-Nitrosamines are environmental contaminants found in food, water, drugs, cosmetics, and tobacco products [1]. Human exposure to *N*-nitrosamines also occurs through nitrosation of amines in the body via acid- or bacteria-catalyzed reactions with nitrite, or through reactions with products of nitric oxide generated during inflammation or infection [2]. Many *N*-nitroso compounds are mutagenic and carcinogenic in all animal species tested, and are suspected to be carcinogenic to humans [3]. *N*-Methyl-*N*-nitrosourea (MNU) is an *N*-nitroso compound that can be formed in the body [4, 5]. MNU is a direct-acting alkylating agent that reacts with DNA to produce DNA adducts, such as *O*^6^-methylguanine (*O*^6^-MeG) and *N*^3^-methyladenine (*N*^3^-MeA) [6, 7]. *O*^6^-MeG can cause miscoding errors during DNA replication, leading to GC-to-AT transitions. *N*^3^-MeA is not considered a serious promutagenic lesion, based on studies performed in bacterial and yeast systems. In B-form DNA, the right-handed helical structure creates distinct major and minor grooves that differ markedly in their chemical accessibility to alkylating agents. The *O*^6^ position of guanine projects into the major groove [8], whereas the *N*^3^ position of adenine lies in the minor groove [9].

Carcinogenesis may be initiated by the formation of adducts between carcinogens and DNA, followed by mutations or DNA damage. Therefore, preventing the initial step of chemical binding of carcinogens to DNA using edible chemopreventive agents is an important strategy for cancer prevention [10].

**Fig. 1:**
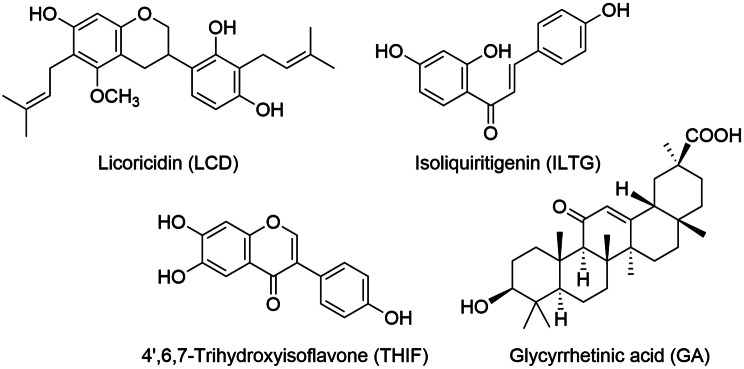
Chemical structures of the flavonoids used in this study

Flavonoids are widely recognized for their beneficial health effects, including antiviral, antiproliferative, antiallergic, anticancer, and anti-inflammatory activities [11]. Epidemiological studies have shown that diets rich in flavonoids, such as fruits, vegetables, and beverages, protect against cancer [12]. We previously reported that flavonoids from several plant species of the *Leguminosae* family have antimutagenic effects on MNU-induced mutagenicity [13, 14]. Because licoricidin (LCD, an isoflavan) [15] and isoliquiritigenin (ILTG, a chalcone) [16] showed high antimutagenic activity among the tested compounds, in this study, we aimed to elucidate their antimutagenic mechanisms against MNU (Fig. 1). In contrast, 4′,6,7-trihydroxyisoflavone (THIF) and glycyrrhetinic acid (GA), which do not show any antimutagenic activity against MNU, were used as negative controls.

Although the DNA interactions of small molecules, such as quercetin, resveratrol, genistein, and epigallocatechin gallate, are well known, modes of their binding to DNA and their functional roles in the genome remain unclear [12, 17–19]. The minor-groove binder distamycin qualitatively inhibits the production of minor-groove adducts when DNA is treated with MNU or methyl methanesulfonate; however, few studies have shown that this inhibition correlates with the antimutagenic activity against MNU [20]. Therefore, we aimed to clarify the antimutagenic mechanism of these flavonoids by evaluating their noncovalent interactions with calf thymus DNA (ctDNA) using a UV-Vis spectrophotometer and quantifying the DNA adducts (*O*^6^-MeG and *N*^3^-MeA) in a mixture of the compounds and MNU. Additionally, we analyzed the half-lives of MNU and the amount of flavonoids remaining in the reaction mixture to elucidate the direct reaction between the flavonoids and MNU. Figure 1 shows the structures of the flavonoids used in this study.

## Materials and methods

### Materials

ILTG and THIF were obtained from Tokyo Chemical Industry Co., Ltd. (Tokyo, Japan). Hoechst 33258 was purchased from Nacalai Tesque (Kyoto, Japan). *N*^3^-MeA and *O*^6^-MeG were obtained from Sigma–Aldrich (St. Louis, MO, USA). Sodium ammonium hydrogen phosphate tetrahydrate was obtained from Merck (Darmstadt, Germany). Bacto agar and Bacto nutrient broth were obtained from Becton Dickinson Microbiology Systems (Sparks, USA). All other reagents were obtained from FUJIFILM Wako Pure Chemical Co., Ltd. (Osaka, Japan). MNU [mp. 106 °C (decomp.)] was prepared as described previously [21]. LCD was isolated from a powdered ethanolic extract of *Glycyrrhiza aspera* (China) root (Chiba, Japan; Tokiwa Phytochemical Co., Ltd.). The purities of the synthesized MNU, LCD, ILTG, GA, and THIF were determined from their ^1^H-NMR spectra, where all the required integral values matched the number of protons without any other peak.

The ctDNA was purified via phenol-chloroform and ethanol precipitation. An absorbance ratio (A_260_/A_280_) greater than 1.8 indicated sufficient DNA purity and absence of proteins. DNA concentration was calculated using the standard conversion that an absorbance of 1.0 at 260 nm corresponds to 50 μg/mL of double-stranded DNA [22]. Its stock solution was prepared in TE buffer and stored at 4 °C.

### UV absorption studies

The UV absorption spectra were recorded using an Agilent 8453 spectrophotometer equipped with an Agilent 89090A thermostat. For LCD, TE buffer (2988 μL) and test compound (5 mM, 12 μL acetonitrile; final concentration 20 μM) were added to a 1.0 cm quartz cell. After thorough mixing, the mixture was titrated with ctDNA (4.1 mM, 2 μL per addition at 5 min intervals), resulting in final ctDNA concentrations of 2.72–27.2 μM. For other compounds, TE buffer (2988 μL) and test compound (5 mM, 12 μL dimethyl sulfoxide (DMSO); final concentration 20 μM) were added to a 1.0 cm quartz cell [23, 24]. After thorough mixing, the mixture was titrated with ctDNA (0.82 mM, 10 μL per addition at 5 min intervals), resulting in final ctDNA concentrations of 2.72–26.4 μM. UV spectra were measured and recorded over a wavelength range from 200 to 600 nm at 293, 298, 303, 308, 310, and 313 K using the TE buffer solution as a reference. The experiments were repeated three times to confirm reproducibility. The value of binding constant (*K*, 298 K, pH 8.0, 13.4 µM DNA) was determined using Eq. (1) [23, 24].

$$\matrix{ {{1 \over {{\bf{\it{A}}} - {{\bf{\it{A}}}_0}}} = {1 \over {{{\bf{\it{A}}}_\infty } - {{\bf{\it{A}}}_0}}} + {1 \over {\left. {{{\left( {\bf{\it{A}}} \right.}_\infty } - {{\bf{\it{A}}}_0}} \right) \cdot {\bf{\it{K}}}}} \cdot {1 \over {{{\bf{\it{C}}}_{{\bf{\it{DNA}}}}}}}} \cr } \left( 1 \right){\bf{\it{ }}}$$

where, A_0_ and A are the absorbance of compound in the absence and presence of ctDNA, respectively. A_∞_is the absorbance of compound with the maximum concentration of DNA.

*K* at five temperatures were obtained and used to calculate the thermodynamics parameters, Gibbs free energy (*ΔG*), enthalpy change (*ΔH*), and entropy change (*ΔS*). Gibbs free energy (*ΔG*) was calculated using Eq. (2) [23].

2 $$\Delta G = - RTlnK$$

*ΔH* and *ΔS* were obtained from slopes and intercepts of the plots of ln *K* versus 1000/T, respectively, using the van’t Hoff equation (Eq. (3)) [23].

3 $$\matrix{ {lnK = - {{\Delta {\bf{\it{H}}}} \over {{\bf{\it{RT}}}}} + {{\Delta {\bf{\it{S}}}} \over {\bf{\it{R}}}}} \cr } $$

### Bacterial mutation assay

Antimutagenic effect of each compound was assayed according to the Ames method using a plate incorporation protocol [25, 26]. *S. typhimurium* TA1535 was kindly provided by Professor B. N. Ames (University of California, Berkeley, USA).

MNU solution (1.5 μmol/50 μL of DMSO) was added to a test tube, followed by 0.5 mL of 0.1 M sodium phosphate buffer (pH 7.4), 50 μL of each test compound solution in DMSO, and 0.1 mL *S. typhimurium* TA1535 culture. The mixture was thoroughly mixed. Next, 2 mL of top agar was added, and the mixture was poured onto a minimal glucose agar plate. Revertant colonies were counted after incubation at 37 °C for 44 h. Plates without MNU or the test compound were used as negative controls. All tested plates were microscopically examined for thinning or for the absence of a background lawn and microcolony formation, which are considered indicators of toxicity induced by the test material. Neither MNU nor the compounds displayed toxicity against *S. typhimurium* TA1535 in the antimutagenicity test (Tables S3–S8).

Mutagenic activity in the presence of compounds was expressed as a percentage of mutagenicity (% = Rs/R × 100), where Rs is the number of His^+^ revertants per plate exposed to MNU and compounds, and R is the number of His^+^ revertants per plate with MNU alone. The number of spontaneous revertants was subtracted to obtain the Rs and R values. The mutagenicity of MNU alone was defined as 100%. The experiments were repeated three times, and similar results were obtained.

Toxicity assays were performed under the same conditions as those used for the Ames test to determine the maximum concentrations of the compounds that could be added without exerting toxic effects on the bacteria. MNU solution (1.5 μmol/50 μL of DMSO) was added to the test tube, followed by 0.5 mL of 0.1 M sodium phosphate buffer (pH 7.4), 50 μL of each compound concentration, and 0.1 mL *S. typhimurium* TA1535 culture. A portion of the mixture was diluted 10^5^ times in 1/15 M phosphate buffer (pH 7.4). Histidine-free top agar (2 mL) was added to 200 μL of the diluted solution, and the resulting mixture was poured onto an NB agar plate. The colonies were counted after incubation at 37 °C for 20 h. A substance was considered cytotoxic when the bacterial survival rate was < 80%, as observed for the negative control. Mutation frequency was determined by calculating the number of mutants per 10^7^ surviving bacterial cells. The experiments were performed in triplicate (*n* = 3) and repeated three times to confirm reproducibility, and the data are expressed as mean ± SD.

### Analysis of *O*^6^-MeG and *N*^3^-MeA formation in ctDNA

Test compound solutions were prepared in DMSO at concentrations of 0, 0.1 µmol/100 µL (final concentration 0.2 mM), 0.5 µmol/100 µL (final concentration 1.0 mM), 1.0 µmol/100 µL (final concentration 2.0 mM), 2.0 µmol/100 µL (final concentration 4.0 mM), and 4.0 µmol/100 µL (final concentration 8.0 mM). MNU solution was freshly prepared in DMSO at a concentration of 150 nmol/50 µL. A test compound solution (100 µL) was added to the mixture of DNA solution (0.15 mg/mL, 250 µL, final concentration 0.1 mM) and H_2_O (115 µL), and incubated at 37 °C for 2 h. Then, MNU solution (35 µL, final concentration 0.2 mM) was added to the reaction mixture and incubated at 37 °C for 16 h (total volume 500 µL/tube). The solution was divided into two tubes. To precipitate the DNA, 3 M ammonium acetate (25 µL) and ethanol (500 µL) were added to the mixture, which was subsequently placed overnight in a − 30 °C freezer. The solutions were subsequently centrifuged at 15,000 rpm for 15 min at 4 °C, after which the supernatant was removed. The DNA was dissolved in diluted TE [100 µL, 3 mM Tris-HCl (pH 8.0) + 0.2 mM EDTA (pH 8.0)] in a hot water bath at 55 °C for 45 min. Three molar sodium acetate (10 µL) and ethanol (500 µL) were added to the mixture and cooled at − 30 °C for 2 h, and the supernatant obtained after centrifugation was removed. The precipitated DNA was redissolved in diluted TE and centrifuged twice to wash the DNA two more times. After the supernatant was removed, 70% ethanol (400 µL) was added, and the mixture was subsequently centrifuged at 15,000 rpm for 15 min at 4 °C. The supernatant was removed, and the residue was evaporated to dryness under vacuum. The isolated DNA pellet was redissolved in water (47.5 µL) in a hot water bath (55 °C), and DNA solution from the two tubes were combined. Formic acid (5 µL) was added to the DNA mixture, which was subsequently heated at 85 °C for 60 min. The resulting mixture (1 µL) was injected into the HPLC-UV-MS instrument. The analysis was performed using an Agilent 6470 Triple Quadrupole LC/MS system interfaced with an Agilent 1260 series HPLC system. Electrospray ionization was performed in positive mode. Mass transitions (precursor-to-product) monitored were 166.1 > 149.0 for *O*^6^-MeG [27] and 150.0 > 123.0 for *N*^3^-MeA [28]. The LC and MS parameters are listed in the Additional file 1 (Tables S9 and S10). The experiments were performed in triplicate (*n* = 3) and repeated two times to confirm reproducibility, and the data are expressed as mean ± SD.

### Half-lives of MNU

MNU (0.75 μmol/25 μL acetonitrile, final concentration 2.1 mM) and test compound (1.0 µmol/25 µL acetonitrile, final concentration 2.9 mM) were added to 0.1 M phosphate buffer (250 µL) and acetonitrile (50 µL). The reaction was initiated by adding MNU. The mixture was then incubated at 25 °C for 2 h. An aliquot of the mixture was diluted 15-fold with acetonitrile at specified intervals, and the solution (2 μL) was injected into the HPLC system. Acetonitrile (25 µL) was used as a control instead of the test compound. The instrument used for HPLC-UV analysis consisted of a pump (LC-6AD, Shimadzu, Kyoto, Japan), a UV detector (SPD-20A, Shimadzu), an ODS column (Mightysil RP-18GP, 4.6 × 250 mm, 5 µL, Kanto Chemical Co., Inc., Tokyo, Japan), a column oven (CTO-20A, Shimadzu), and a recorder (C-R8A, Shimadzu). A methanol:water (1:9) mixture was used as the mobile phase. The flow rate of the mobile phase, column temperature, and detection wavelength were set to 0.5, 25 °C, and 254 nm, respectively. The experiments were repeated four times to confirm reproducibility, and the data are expressed as mean ± SD (*n* = 4).

### Quantification of the remaining test compounds

The reaction mixture was prepared as described for the measurement of the half-life of MNU. MNU (0.75 μmol/25 μL acetonitrile, final concentration 2.1 mM) and a test compound (1.0 µmol/25 µL acetonitrile, final concentration 2.9 mM) were added to 0.1 M phosphate buffer (250 µL) and acetonitrile (50 µL). The reaction was initiated by adding MNU. Acetonitrile (25 µL) was used instead of MNU as a control. The mixture was then incubated at 25 °C for 24 h, and 2 µL of the mixture was analyzed using HPLC. The experiments were repeated four times to confirm reproducibility, and the data are expressed as mean ± SD (*n* = 4). The HPLC conditions for each compound were as follows:

**Separation of the LCD sample.** Column: Mightysil, RP-18 GP, 4.6 mm × 250 mm (5 μm); eluent: methanol: water (75:25); flow rate: 1.0 mL/min; detection wavelength: 254 nm.

**Separation of the ILTG sample.** Column: Mightysil, RP-18 GP, 4.6 mm × 250 mm (5 μm); eluent: methanol: water (60:40); flow rate: 1.0 mL/min; detection wavelength: 323 nm.

**Separation of the THIF sample.** Column: Capcell pak, UG120 2.0 mm × 150 mm (5 μm); eluent: acetonitrile: water (15:85); flow rate: 0.5 mL/min; detection wavelength: 360 nm.

**Separation of the GA sample.** Column: Mightysil, RP-18 GP, 4.6 mm × 250 mm (5 µm); eluent: methanol: 0.3% aqueous acetic acid (90:10); flow rate: 0.5 mL/min; detection wavelength: 250 nm. GA was dissolved in tetrahydrofuran instead of acetonitrile.

## Results

### DNA binding affinity

Absorption spectroscopy is a convenient technology that is widely used to study interactions between small molecules and ctDNA. Small molecules can interact with DNA through the following three noncovalent modes: (i) electrostatic interactions with the anionic sugar–phosphate backbone of DNA, (ii) groove binding in the DNA groove, and (iii) intercalation between base pairs [24]. The absorption bands of the complexes were affected by the increasing concentrations of ctDNA. To investigate the mode of interaction between ctDNA and flavonoids, we used intercalative ethidium bromide (EtBr) and minor- groove binding Hoechst 33258 as positive controls.

The absorption spectra of LCD, ILTG, EtBr, and Hoechst 33258, when titrated with ctDNA, showed isosbestic points at 240, 305, 272, and 370 nm, respectively (Fig. S1). These data provide evidence of compound–DNA complex formation. For THIF and GA, the interaction with DNA did not affect the absorbance or UV maximum of the compounds with increasing DNA concentration, indicating that the compounds did not interact with DNA. The binding constant *K* (M^−1^) values at 298 K were in the following order: Hoechst 33258 (3.78 × 10^5^) >>LCD (1.19 × 10^5^) >ILTG (0.83 × 10^5^) ≓ EtBr (0.81 × 10^5^) (Figs. S2–S5).

**Fig. 2:**
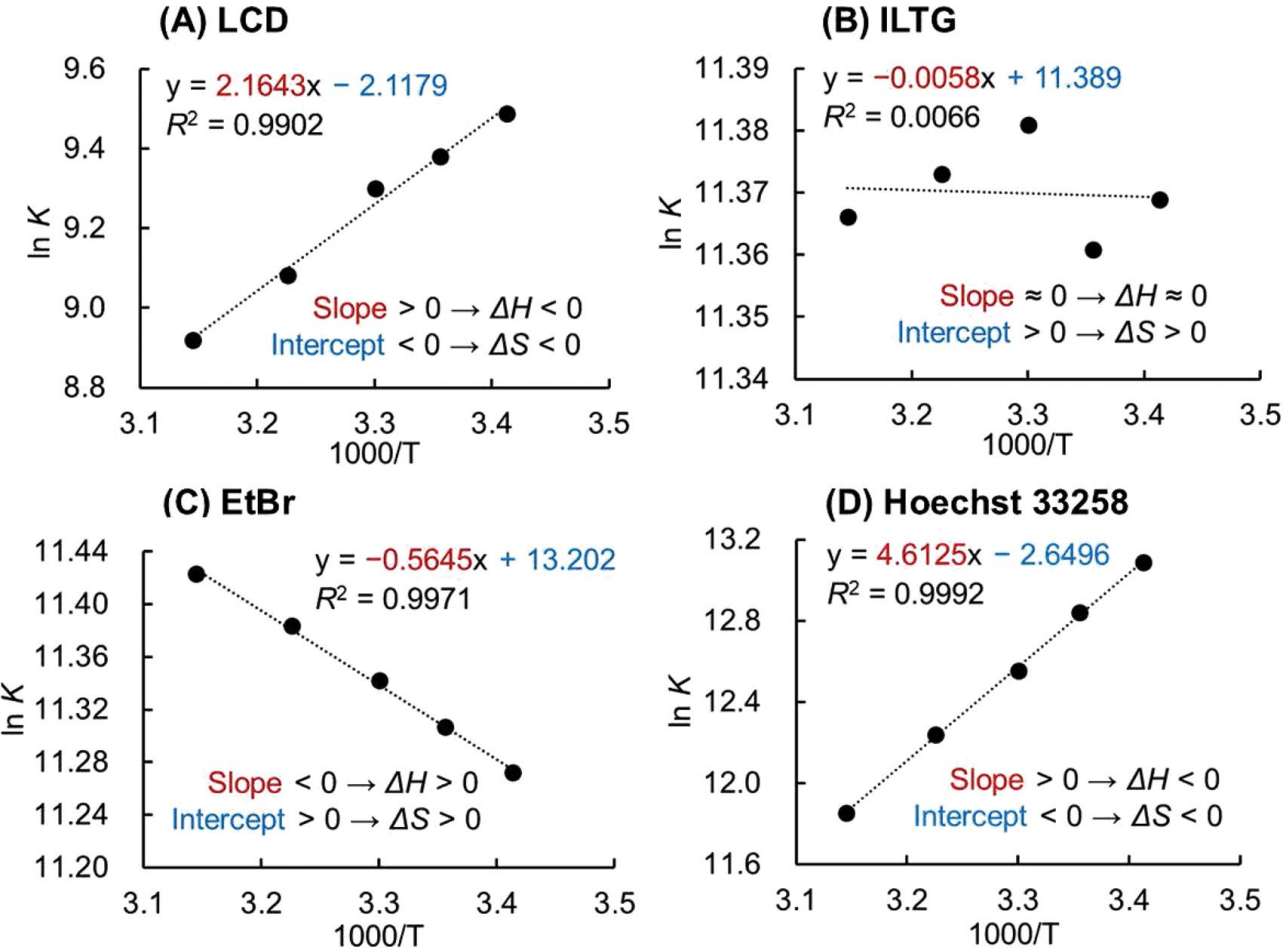
van’t Hoff plot for determination of the thermodynamic parameters of compound–DNA

The thermodynamic parameters, *ΔG*, *ΔH*, and *ΔS*, can be used to elucidate the interactions between a small molecule and DNA [23, 24]. The mode of interaction of the compounds was determined as reported previously [29, 30]. For EtBr binding to DNA, *ΔH* and *ΔS* were > 0, which implied intercalative interaction (Fig. 2, Table 1). In contrast, the negative values of *ΔH* and *ΔS* for Hoechst 33258 and LCD implied a minor-groove interaction. For ILTG, with *ΔH* ≈ 0 and *ΔS* > 0, the major force was electrostatic interaction. All *ΔG* values were negative, indicating that the compounds bound to ctDNA spontaneously.

**Table 1:** Thermodynamic parameters of the test compounds

	*ΔH* (kJ/mol)	*ΔS* (J/mol)	*ΔG* (kJ/mol) at 298 K
LCD	−18.0	−17.6	−28.9
ILTG	0.05	94.6	−28.0
EtBr	4.7	109.7	−28.0
Hoechst 33258	−38.3	−22.0	−31.8

TE buffer and test compound were added to a 1.0 cm quartz cell. The mixture was mixed thoroughly and titrated with ctDNA. UV spectra were measured and recorded from 200 to 600 nm at each temperature using the TE buffer solution as a reference

### Mutagenicity of MNU in the presence of flavonoids

**Fig. 3:**
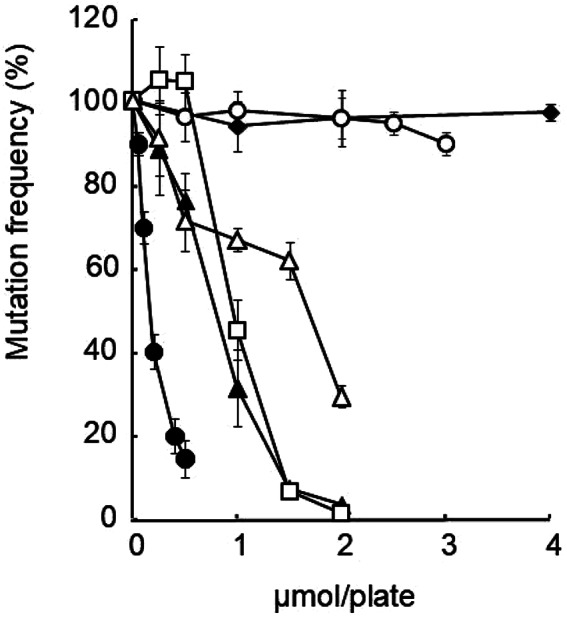
Mutation frequency of MNU in the presence of noncovalent DNA binders in *Salmonella typhimurium* TA1535 the SD bars were too small to show. ●: LCD, ▲: ILTG, ◆: THIF, 〇: GA, □: EtBr, △: Hoechst 33258. Error bars indicate SD (*n* = 3). Some error bars are too small to show

In a previous study, we focused on isolating antimutagens from plants and used microgram units for the Ames assay. In this study, we used the Ames assay with mol units to measure the antimutagenic activity of LCD and ILTG against MNU (Fig. 3, Tables S3–S8). EtBr and Hoechst 33258 were also assayed for their antimutagenic activity against MNU (1.5 µmol/plate). THIF was used as a negative control. LCD, ILTG, and Hoechst 33258 suppressed MNU-induced mutagenicity at increasing concentrations of the compounds, without any cytotoxicity. THIF (1.0–8.0 µmol/plate) did not affect the mutagenicity of MNU (Table S5). EtBr enhanced MNU-induced mutagenicity at lower concentrations (0.25–0.5 µmol/plate) and then decreased mutagenicity at higher concentrations (1.0–2.0 µmol/plate). The half-maximal inhibitory (IC_50_) values of the test compounds were as follows: 0.15, 0.8, 1.0, and 1.9 µmol/plate for LCD, ILTG, EtBr, and Hoechst 33258, respectively.

### Quantification of DNA adducts derived from MNU

**Fig. 4:**
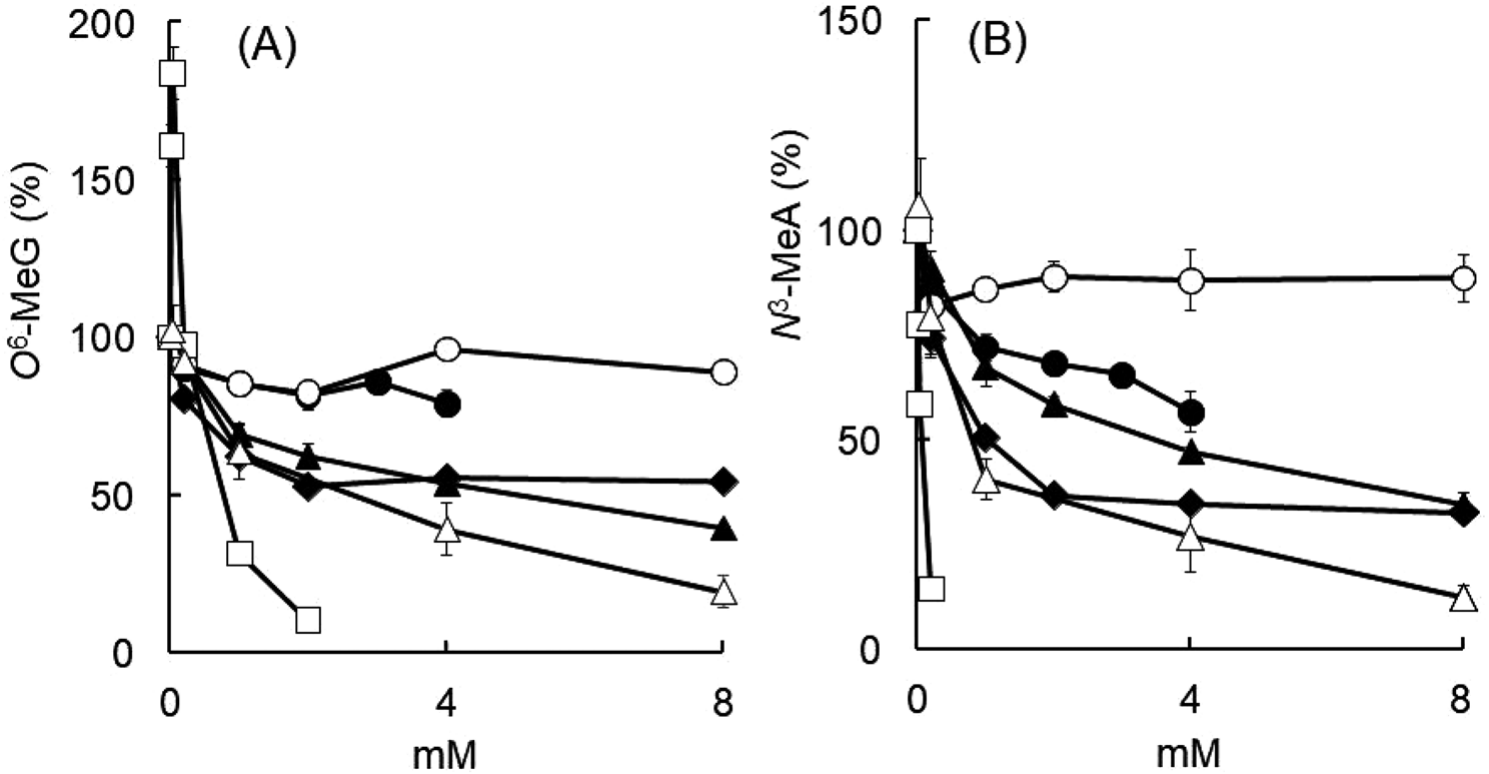
Quantification of *O*^6^-MeG (**A**) and *N*^3^-MeA (**B**) in a reaction mixture of noncovalent DNA binders and MNU. ●: LCD, ▲: ILTG, ◆: THIF, 〇: GA, □: EtBr, △: Hoechst 33258. The incubation mixture contained DNA solution (0.1 mM) and the test compound (0.02 mM–8.0 mM). After the mixture was incubated at 37 °C for 2 h, MNU solution (0.2 mM) was added to the mixture and incubated at 37 °C for 16 h. Error bars indicate SD (*n* = 3). Some error bars are too small to show

To elucidate the mechanisms for the inhibition of MNU-induced mutagenicity by LCD and ILTG, *O*^6^-MeG and *N*^3^-MeA were quantified by treating ctDNA with MNU in the presence of flavonoids (Fig. 4). EtBr, Hoechst 33258, GA, and THIF were used as controls. The test compounds did not affect the decomposition of *O*^6^-MeG and *N*^3^-MeA during the reaction or upon acidic hydrolysis of DNA (Tables S11 and S12).

THIF decreased *O*^6^-MeG and *N*^3^-MeA levels to a similar extent, whereas GA did not affect the levels of either DNA adduct (Fig. 4, Table S15, S16). *N*^3^-MeA decreased with increasing EtBr concentration. *O*^6^-MeG increased with increased concentration of EtBr (0.02–0.04 mM), followed by a decrease at higher concentrations (0.2–2.0 mM) (Fig. 4, Table S17). Hoechst 33258 was more effective at reducing *N*^3^-MeA levels at low concentrations, whereas at 8 mM it inhibited *O*^6^-MeG and *N*^3^-MeA to a similar extent (Fig. 4, Table S18). LCD preferentially reduced *N*^3^-MeA at all the tested concentrations; at 4 mM, *O*^6^-MeG and *N*^3^-MeA levels were reduced to 79 and 57%, respectively (Fig. 4, Table S13). ILTG caused comparable reduction in the levels of *O*^6^-MeG and *N*^3^-MeA at all the concentrations tested; at 8 mM ILTG, *O*^6^-MeG and *N*^3^-MeA levels were reduced to 40 and 35%, respectively (Fig. 4, Table S14).

### Direct reaction of MNU and flavonoids

To investigate the direct interaction between MNU and the flavonoids, we used HPLC to measure the half-life of MNU and to determine the amount of flavonoid remaining in the reaction mixture. The half-life of MNU was similar in the presence and absence of LCD, ILTG, and Hoechst 33258 (Table 2, Table S19–S22, Fig. S6). The half-life of MNU decreased in the presence of THIF and EtBr, indicating that the compounds decomposed MNU.

**Table 2:** Half-lives of MNU in the presence of the test compounds

	Half-life of MNU (min)
Control	25.0 ± 0.5
LCD	25.0 ± 1.8
ILTG	24.6 ± 0.6
THIF	16.2 ± 1.1
GA	30.0 ± 2.1
EtBr	15.6 ± 0.9
Hoechst 33258	23.8 ± 1.1

The incubation mixture contained MNU (2.1 mM), the test compound (2.9 mM), and 0.1 M phosphate buffer (pH 7.4). Acetonitrile was used as a control instead of MNU. The mixture was incubated at 25 °C for various durations

The amounts of LCD and ILTG did not change, and no new peaks were detected in the reaction mixture of MNU and flavonoids over 24 h (Table 3, Table S23–S26). These data demonstrate that LCD and ILTG did not react directly with MNU and did not scavenge the reactive intermediate methyldiazonium ions derived from MNU. In the presence of MNU, THIF decreased in a time-dependent manner, and the half-life of MNU was significantly shortened.

**Table 3:** Remaining (%) of the test compounds

Time	LCD		ILTG		THIF		GA	
(hours)	+MNU	- MNU	+MNU	- MNU	+MNU	- MNU	+MNU	- MNU
0	100.0	100.0	100.0	100.0	100.0	100.0	100.0	100.0
1	99.8	100.1	101.7	97.3	91.0	97.0	101.7	93.2
6	96.8	93.6	103.4	95.1	82.5	100.5	102.6	100.4
24	91.5	91.4	95.6	95.2	75.9	95.8	101.9	102.2

The incubation mixture contained MNU (2.1 mM), the test compound (2.9 mM) and 0.1 M phosphate buffer (pH 7.4). Acetonitrile was used as a control instead of MNU. The mixture was incubated at 25 °C for various durations

## Discussion

GA failed to show antimutagenic activity against MNU and did not affect the number of DNA adducts, suggesting that it did not interact directly with DNA (Figs. 3, 4, S1 and Table S16).

THIF (2.9 mM) reduced the half-life of MNU (2.1 mM) (Table 2). For quantification of the DNA adducts, the concentration of THIF used (4.0 mM) was approximately 19-fold higher than that of MNU (0.2 mM). Under these conditions, THIF-mediated decomposition of MNU led to a marked reduction in the formation of both *O*^6^-MeG and *N*^3^-MeA (Fig. 4).

In the EtBr experiments, *O*^6^-MeG levels increased 1.8-fold at 0.01 µmol of EtBr, followed by a decrease at 0.05 µmol of EtBr (Fig. 4). These changes in DNA adduct formation showed the same trend as the mutagenicity observed in *S. typhimurium* TA1535 (Fig. 3). At low concentrations of EtBr, the interaction of EtBr with the DNA duplex can occur through both the minor and major grooves; however, EtBr is very weakly bound to the major groove [31]. Thus, EtBr may interact with the minor groove side at low concentrations, selectively inhibiting the methylation of the *N*^3^ position of adenine, resulting in a relative increase in alkylation at the *O*^6^ position. At high concentrations, Hoechst 33258 and EtBr reduced *O*^6^-MeG and *N*^3^-MeA levels to a similar extent. The minor-groove binder distamycin strongly inhibits the formation of *N*^3^-MeA in DNA exposed to methylating agents but only slightly decreases the levels of *N*^7^-MeG [20]. At high concentrations, Hoechst 33258 and EtBr interfered with the formation of *O*^6^-MeG and *N*^3^-MeA through nonspecific interactions with the DNA sugar–phosphate backbone due to their cationic nature. Additionally, the binding of dyes may induce changes in DNA conformation, thereby reducing the accessibility of alkylating agents [32, 33]. Although Hoechst 33258 and EtBr substantially decreased DNA adduct formation, they showed lower mutagenic activity in *Salmonella* strains than did LCD and ILTG, possibly because their cationic nature limits permeability across bacterial cell membranes.

Spectroscopic and thermodynamic analyses clearly indicated that both LCD and ILTG exhibit high binding affinities for ctDNA. Notably, LCD functions as a minor-groove binder, whereas ILTG interacts mainly with the DNA phosphate–sugar backbone, suggesting distinct binding modes for the two compounds (Table 1). Neither LCD nor ILTG affected the decomposition of MNU, as shown in Table 2. Consistently, no significant changes were observed in the levels of LCD or ILTG during the reaction with MNU (Table 3). These observations suggested that LCD and ILTG act as antimutagens rather than desmutagens.

The preferential binding of LCD to the DNA minor groove more effectively suppresses the formation of *N*^3^-MeA than that of *O*^6^-MeG, consistent with the effects observed for the mino-groove binder Hoechst 33258 (Fig. 4). In contrast, ILTG may interact with the DNA sugar–phosphate backbone, potentially resulting in a comparable reduction in the levels of both adducts (Fig. 4). Despite LCD exhibiting greater antimutagenic activity and DNAbinding capacity, ILTG exhibited a stronger inhibitory effect on DNA adduct formation. Because MNU-induced mutagenesis in *S. typhimurium* TA1535 is mainly attributed to the formation of *O*^*6*^-MeG [34, 35], the antimutagenic effect was presumed to involve additional mechanisms. The antimutagenic activity of ILTG may, therefore, be partly explained by its interaction with DNA, leading to a decrease in *O*^*6*^-MeG formation. In contrast, the antimutagenic effects observed for LCD were likely attributable to alternative mechanisms independent of *O*^*6*^-MeG reduction. These findings suggested that interactions between flavonoids and DNA may contribute to the modulation of gene expression and/or the alteration in enzyme activity, either directly or indirectly [36]. These enzymes participate in (i) the accurate replication of damaged bases by inserting correct nucleotides, and (ii) the promotion error-free DNA repair pathways, or the suppression of error-prone enzymes [37–40]. Although mechanisms underlying the antimutagenic effect of flavonoids in the *Salmonella* assay have been reported to involve inhibition of enzymatic activation, direct-acting mutagens were used in this study suggested that the antimutagenicity of flavonoids is not associated with the metabolic activation system [41]. MNU treatment induces DNA alkylation and may also be associated with increased intracellular levels of reactive oxygen species (ROS) [42, 43]. Flavonoids are known to possess radical-scavenging activity as well as metal-chelating properties, which may contribute to the prevention of DNA damage caused by ROS [44–46]. Although the biological activities of flavonoids have been extensively studied, only a limited number of studies have addressed the relationship between their interactions with DNA and their biological activities [47, 48]. Based on spectroscopic and molecular docking studies, Khan et al. proposed that rutin exhibits antigenotoxicity through noncovalent interactions, protecting DNA from chemically induced mutations; however, no data are available on DNA adducts [47]. Our results clearly indicate that one of the mechanisms contributing to the prevention of MNU-induced mutagenicity and DNA methylation involves the binding of flavonoids (LCD and ILTG) to duplex DNA. Furthermore, our data suggest that LCD and ILTG exert their antimutagenic effects through this mechanism, potentially in combination with additional mechanism.

This study had certain limitations. First, the experiments were performed using bacterial and in vitro DNA systems, which may not fully represent the complexity of mammalian cellular environments, including chromatin structure, DNA repair capacity, and metabolic processes. Second, the solubility constraints of certain flavonoids limited the evaluation of higher concentrations that might further influence the formation of DNA adducts. Third, sequence-specific DNA binding preferences were not investigated; therefore, the genomic selectivity of flavonoid–DNA interactions remains unclear.

## Conclusions

The present study demonstrates that LCD and ILTG directly interact with DNA and suppress DNA adduct formation. Although these interactions may contribute to their antimutagenic properties, the data indicate that DNA binding alone does not fully account for their antimutagenic activity, suggesting the involvement of additional mechanisms. Further investigations using cell-based experimental systems will be essential to fully elucidate the underlying mechanisms, as well as to identify preferential DNA sequences that interact with flavonoids and to clarify the structural basis of their antimutagenic activity.

## Electronic supplementary material

Below is the link to the electronic supplementary material.

Supplementary Material 1

## Data Availability

No datasets were generated or analysed during the current study.
