# Supplementary material for: Mechanistic investigation of flavonoid-mediated inhibition of N-methyl-N-nitrosourea-induced mutagenicity via noncovalent DNA binding
Source: Genes Environ. 2026 Jun 10;48:13. doi: 10.1186/s41021-026-00361-3 (PMC13276938; doi:10.1186/s41021-026-00361-3)
Supplement: Supplementary file 1 — Supplementary Material 1 [file 41021_2026_361_MOESM1_ESM.docx]

**Mechanistic investigation of flavonoid-mediated inhibition of *N*-methyl-*N*-nitrosourea-induced mutagenicity via noncovalent DNA binding**

Chisato Matsuoka, Nao Takatama, Takuya Muraoka and Keiko Inami*

Contents

| **Figure S1** | UV-Visible spectra of the test compounds with increasing DNA concentration | **S1** |
| --- | --- | --- |
| **Figure S2** | Noncovalent binding constants for licoricidin-DNA complex at various temperature | **S2** |
| **Figure S3** | Noncovalent binding constants for isoliquiritigenin-DNA complex at various temperature | **S3** |
| **Figure S4** | Noncovalent binding constants for ethidium bromide-DNA complex at various temperature | **S4** |
| **Figure S5** | Noncovalent binding constants for Hoechst 33258-DNA complex at various temperature | **S5** |
| **Table S1** | Free energy values for the compound-DNA complexes at various temperature | **S6** |
| **Table S2** | Thermodynamic parameter for the compound-DNA complexes | **S6** |
| **Table S3** | Mutagenicity of licoricidin against MNU in *S. typhimurium* TA1535 | **S6** |
| **Table S4** | Mutagenicity of isoliquiritigenin against MNU in *S. typhimurium* TA1535 | **S7** |
| **Table S5** | Mutagenicity of 4’,6,7-trihydroxyisoflavone against MNU in *S. typhimurium* TA1535 | **S7** |
| **Table S6** | Mutagenicity of glycyrrhetinic acid against MNU in *S. typhimurium* TA1535 | **S8** |
| **Table S7** | Mutagenicity of ethidium bromide against MNU in *S. typhimurium* TA1535 | **S8** |
| **Table S8** | Mutagenicity of Hoechst 33258 against MNU in *S. typhimurium* TA1535 | **S9** |
| **Table S9** | LC instrument conditions for *O^6^*-MeG and *N^3^*-MeA | **S9** |
| **Table S10** | MS instrument conditions for *O^6^*-MeG and *N^3^*-MeA | **S10** |
| **Table S11** | Stability of *O^6^*-MeG and *N^3^*-MeA in the presence of the compounds | **S10** |
| **Table S12** | Stability of *O^6^*-MeG and *N^3^*-MeA under acid hydrolysis conditions | **S11** |
| **Table S13** | Quantification of *O^6^*-MeG and *N^3^*-MeA in the reaction mixture of MNU and licoricidin | **S11** |
| **Table S14** | Quantification of *O^6^*-MeG and *N^3^*-MeA in the reaction mixture of MNU and isoliquiritigenin | **S11** |
| **Table S15** | Quantification of *O^6^*-MeG and *N^3^*-MeA in the reaction mixture of MNU and 4’,6,7-trihydroxyisoflavone | **S12** |
| **Table S16** | Quantification of *O^6^*-MeG and *N^3^*-MeA in the reaction mixture of MNU and glycyrrhetinic acid | **S12** |
| **Table S17** | Quantification of *O^6^*-MeG and *N^3^*-MeA in the reaction mixture of MNU and ethidium bromide | **S12** |
| **Table S18** | Quantification of *O^6^*-MeG and *N^3^*-MeA in the reaction mixture of MNU and Hoechst 33258 | **S13** |
| **Table S19** | Decrease of MNU | **S13** |
| **Table S20** | Decrease of MNU in the presence of licoricidin or isoliquiritigenin | **S13** |
| **Table S21** | Decrease of MNU in the presence of 4’,6,7-trihydroxyisoflavone or glycyrrhetinic acid | **S14** |
| **Table S22** | Decrease of MNU in the presence of ethidium bromide or Hoechst 33258 | **S14** |
| **Figure S6** | Plots of ln (MNU) versus time | **S15** |
| **Table S23** | Quantification of the remaining licoricidin in the presence and absence of MNU | **S16** |
| **Table S24** | Quantification of the remaining isoliquiritigenin in presence and absence of MNU | **S16** |
| **Table S25** | Quantification of the remaining 4’,6,7-trihydroxyisoflavone in the presence and absence of MNU | **S17** |
| **Table S26** | Quantification of the remaining glycyrrhetinic acid in presence and absence of MNU | **S17** |
| **References** |  | **S17** |

**
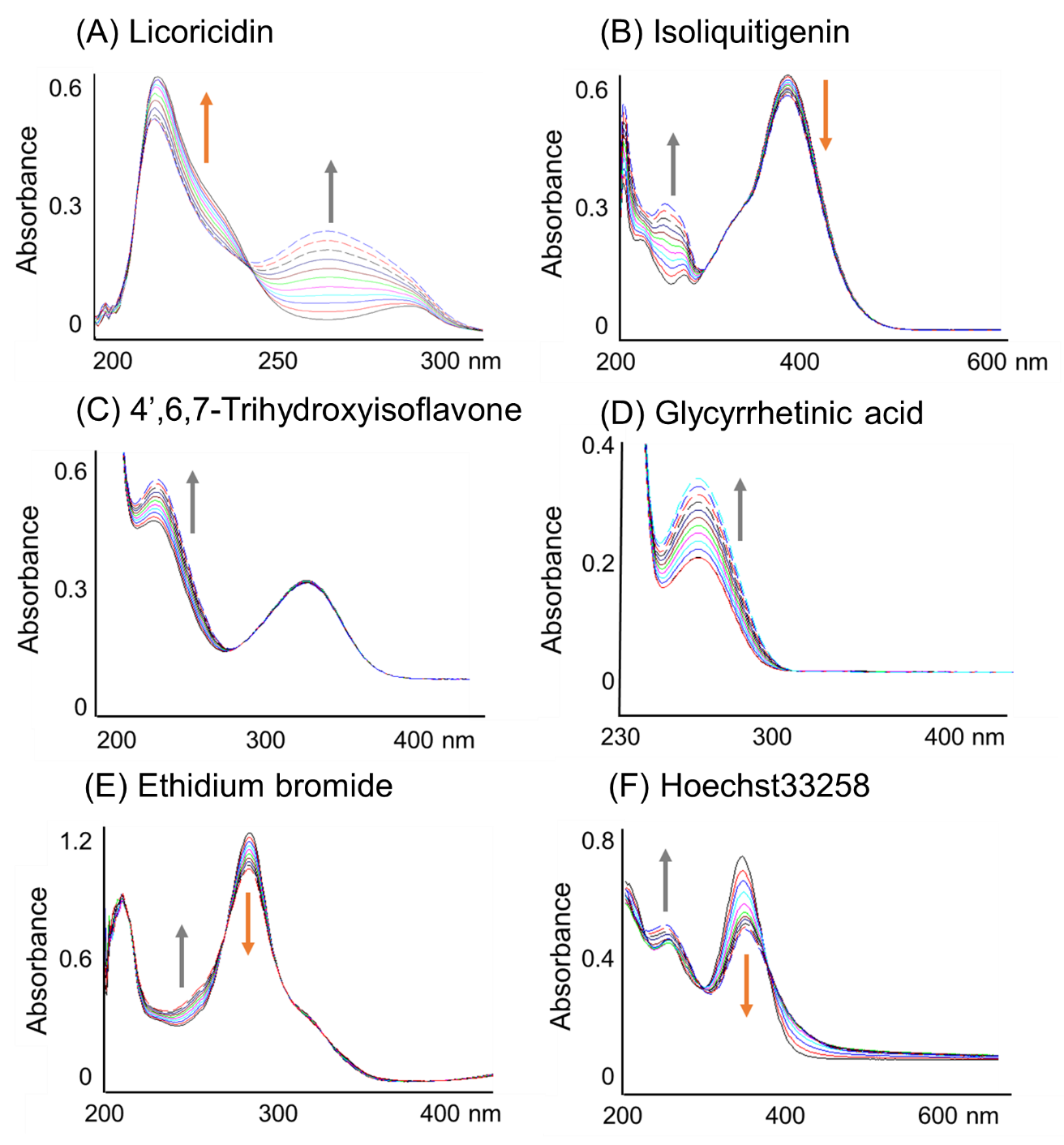
**

**Figure S1**  UV-Visible spectra of the test compounds with increasing DNA concentration

Arrows indicates direction of absorbance changes upon increasing amounts of DNA.

**293 K 298 K**

**303 K 310 K**

**318 K**

| Temp. (K) | Binding constants  (M^-1^) (λ= 210 nm) |
| --- | --- |
| 293 | 1.32×10^5^±0.11 |
| 298 | 1.19×10^5^±0.09 |
| 303 | 1.09×10^5^±0.04 |
| 310 | 8.80×10^4^±0.04 |
| 318 | 7.48×10^4^±0.08 |

**Figure S2 Noncovalent binding constants (M^-1^) for licoricidin-DNA complex at various temperature**

**293 K 298 K**

**303 K 310 K**

**318 K**

| Temp. (K) | Binding constants  (M^-1^) (λ= 395 nm) |
| --- | --- |
| 293 | 8.66×10^4^±0.20 |
| 298 | 8.27×10^4^±0.55 |
| 303 | 8.77×10^4^±0.51 |
| 310 | 8.69×10^4^±0.29 |
| 318 | 8.63×10^4^±0.09 |

**Figure S3 Noncovalent binding constants (M^-1^) for isoliquiritigenin-DNA complex at various temperature**

**293 K 298 K**

**303 K 310 K**

**318 K**

| Temp. (K) | Binding constants (M^-1^) (λ= 285 nm) |
| --- | --- |
| 293 | 7.86×10^4^±0.15 |
| 298 | 8.14×10^4^±0.03 |
| 303 | 8.43×10^4^±0.16 |
| 310 | 8.79×10^4^±0.13 |
| 318 | 9.14×10^4^±0.09 |

**Figure S4 Noncovalent binding constants (M^-1^) for ethidium bromide -DNA complex at various temperature**

**293 K 298 K**

**303 K 310 K**

**318 K**

| Temp. (K) | Binding constants  (M^-1^) (λ= 340 nm) |
| --- | --- |
| 293 | 4.84×10^5^±0.21 |
| 298 | 3.78×10^5^±0.32 |
| 303 | 2.83×10^5^±0.11 |
| 310 | 2.07×10^5^±0.22 |
| 318 | 1.40×10^5^±0.21 |

**Figure S5 Noncovalent binding constants (M^-1^) for Hoechst 33258-DNA complex at various temperature**

**Table S1** Free energy values for compound-DNA complexes at various temperature

| Temp. (K) | *ΔG* (kJ/mol) | | | |
| --- | --- | --- | --- | --- |
|  | licoricidin | isoliquiritigenin | ethidium bromide | Hoechst 33258 |
| 293 | -28.7 | -27.7 | -27.5 | -31.9 |
| 298 | -28.9 | -28.0 | -28.0 | -31.8 |
| 303 | -29.2 | -28.7 | -28.6 | -31.6 |
| 310 | -29.3 | -29.3 | -29.3 | -31.5 |
| 318 | -29.7 | -30.0 | -30.2 | -31.3 |

**Table S2** Thermodynamic parameters for compound-DNA complexes

|  | licoricidin | isoliquiritigenin | ethidium bromide | Hoechst 33258 |
| --- | --- | --- | --- | --- |
| *ΔH* (kJ/mol) | -18.0 | 0.05 | 4.7 | -38.3 |
| *ΔS* (J/mol) | -17.6 | 94.6 | 109.7 | -22.0 |

#### **Table S3** Mutagenicity of licoricidin against MNU in *S. typhimurium* TA1535

| Concentration | Revertants/plate | Survival (%) | | MF |
| --- | --- | --- | --- | --- |
| (µmol/plate) | Colonies ± SE | Colonies ± SE | Survival rate (%) | (%) |
| licoricidin alone 0.25 | 10 ± 3 | – | – | – |
| licoricidin alone 0.5 | 9 ± 3 | – | – | – |
| 0 | 1496 ± 22 | 536 ± 14 | 100.0 | 100.0 |
| 0.05 | 1205 ± 15 | 511 ± 3 | 95.3 | 84.5 |
| 0.1 | 917 ± 51 | 508 ± 41 | 94.8 | 64.7 |
| 0.2 | 494 ± 87 | 480 ± 16 | 89.5 | 36.9 |
| 0.4 | 263 ± 56 | 509 ± 21 | 95.0 | 18.5 |
| 0.5 | 166 ± 24 | 508 ± 20 | 94.8 | 11.7 |
| DMSO | 4 ± 3 | 525 ± 17 | 97.9 |  |

MNU (1.5 µmol/plate)

**Table S4** Mutagenicity of isoliquiritigenin against MNU in *S. typhimurium* TA1535

| Concentration | Revertants/plate | Survival (%) | | MF |
| --- | --- | --- | --- | --- |
| (µmol/plate) | Colonies ± SE | Colonies ± SE | Survival rate (%) | (%) |
| isoliquiritigenin alone 1.0 | 11 ± 2 | – | – | – |
| isoliquiritigenin alone 2.0 | 7 ± 3 | – | – | – |
| 0 | 1517 ± 41 | 503 ± 20 | 100.0 | 100.0 |
| 0.25 | 1336 ± 75 | 501 ± 40 | 99.6 | 89.0 |
| 0.5 | 1175 ± 111 | 513 ± 12 | 102.0 | 75.9 |
| 1.0 | 459 ± 152 | 492 ± 16 | 97.8 | 30.7 |
| 1.5 | 99 ± 22 | 493 ± 26 | 97.9 | 6.6 |
| 2.0 | 44 ± 2 | 503 ± 29 | 99.9 | 2.9 |
| DMSO | 13 ± 1 | 483 ± 20 | 96.0 |  |

MNU (1.5 µmol/plate)

**Table S5** Mutagenicity of 4’,6,7-trihydroxyisoflavone against MNU in *S. typhimurium* TA1535

| Concentration | Revertants/plate | Survival (%) | | MF |
| --- | --- | --- | --- | --- |
| (µmol/plate) | Colonies ± SE | Colonies ± SE | Survival rate (%) | (%) |
| 4’,6,7-trihydroxyiso-flavone alone 4.0 | 11 ± 3 | – | – | – |
| 4’,6,7-trihydroxyiso-flavone alone 8.0 | 9 ± 2 | – | – | – |
| 0 | 1482 ± 42 | 483 ± 24 | 100.0 | 100.0 |
| 1.0 | 1472 ± 94 | 452 ± 9 | 93.7 | 106.0 |
| 2.0 | 1545 ± 101 | 462 ± 19 | 95.6 | 109.0 |
| 4.0 | 1660 ± 36 | 469 ± 15 | 97.1 | 115.3 |
| 6.0 | 1429 ± 60 | 466 ± 26 | 96.5 | 99.9 |
| 8.0 | 1533 ± 31 | 467 ± 35 | 96.8 | 106.9 |
| DMSO | 9 ± 3 | 461 ± 36 | 95.4 |  |

MNU (1.5 µmol/plate)

**Table S6** Mutagenicity of glycyrrhetinic acid against MNU in *S. typhimurium* TA1535

| Concentration | Revertants/plate | Survival (%) | | MF |
| --- | --- | --- | --- | --- |
| (µmol/plate) | Colonies ± SE | Colonies ± SE | Survival rate (%) | (%) |
| glycyrrhetinic acid alone 1.5 | 11 ± 3 | – | – | – |
| glycyrrhetinic acid alone 3.0 | 10 ± 3 | – | – | – |
| 0 | 1270 ± 51 | 495 ± 23 | 100.0 | 100.0 |
| 0.5 | 1290 ± 48 | 523 ± 46 | 105.7 | 96.1 |
| 1.0 | 1218 ± 13 | 486 ± 24 | 98.2 | 97.6 |
| 2.0 | 1220 ± 16 | 488 ± 20 | 98.6 | 95.8 |
| 2.5 | 1223 ± 32 | 503 ± 30 | 101.8 | 94.6 |
| 3.0 | 1085 ± 59 | 471 ± 15 | 95.1 | 89.7 |
| DMSO | 14 ± 2 | 544 ± 24 | 109.9 |  |

MNU (1.5 µmol/plate)

**Table S7** Mutagenicity of ethidium bromide against MNU in *S. typhimurium* TA1535

| Concentration | Revertants/plate | Survival (%) | | MF |
| --- | --- | --- | --- | --- |
| (µmol/plate) | Colonies ± SE | Colonies ± SE | Survival rate (%) | (%) |
| 0 | 14 ± 5 | 527 ± 5 | 100.0 | – |
| ethidium bromide alone 1.0 | 5 ± 1 | 504 ± 5 | 95.5 | – |
| ethidium bromide alone 2.0 | 2 ± 0 | 533 ± 20 | 101.1 | – |
| 0 | 1546 ± 60 | 476 ± 15 | 100.0 | 100.0 |
| 0.25 | 1687 ± 36 | 496 ± 8 | 104.3 | 104.6 |
| 0.5 | 1729 ± 99 | 509 ± 10 | 107.0 | 104.5 |
| 1.0 | 699 ± 103 | 482 ± 46 | 101.3 | 44.6 |
| 1.5 | 91 ± 17 | 460 ± 39 | 96.9 | 6.1 |
| 2.0 | 10 ± 3 | 454 ± 41 | 95.4 | 0.7 |
| DMSO | 14 ± 5 | 490 ± 29 | 102.9 |  |

MNU (1.5 µmol/plate)

**Table S8** Mutagenicity of Hoechst 33258 against MNU in *S. typhimurium* TA1535

| Concentration | Revertants/plate | Survival (%) | | MF |
| --- | --- | --- | --- | --- |
| (µmol/plate) | Colonies ± SE | Colonies ± SE | Survival rate (%) | (%) |
| Hoechst 33258 alone 1.0 | 12 ± 2 | – | – | – |
| Hoechst 33258 alone 2.0 | 9 ± 2 | – | – | – |
| 0 | 1517 ± 38 | 437 ± 19 | 100.0 | 100.0 |
| 0.25 | 1364 ± 46 | 434 ± 10 | 99.2 | 90.7 |
| 0.5 | 1021 ± 87 | 414 ± 7 | 94.7 | 71.1 |
| 1.0 | 890 ± 33 | 386 ± 9 | 88.3 | 66.5 |
| 1.5 | 783 ± 7 | 367 ± 9 | 84.0 | 61.4 |
| 2.0 | 363 ± 33 | 364 ± 12 | 83.2 | 28.8 |
| DMSO | 8 ± 2 | 450 ± 16 | 103.0 |  |

MNU (1.5 µmol/plate)

**Table S9** LC Instrument conditions for *O^6^*-MeG and *N^3^*-MeA

| LC | Agilent Technologies 1260 Infinity II System |
| --- | --- |
| Analytical column | Phenomenex Luna HILIC, 4.6 mm×150 mm (5 µm) |
| Column temperature | 40°C |
| Injection volume | 1 µL |
| Mobile phase | A) 80% Water containing 0.1% formic acid |
|  | B) 20% Methanol containing 0.1% formic acid |
| Flow rate | 0.2 mL/min |
| Stop time | 10 min |
| Post time | 10 min |
| Detection | Guanine, Adenine; 260 nm |

**Table S10** MS Instrument conditions for *O^6^*-MeG and *N^3^*-MeA ^1,2)^

| MS | Agilent 6470 Triple quadruple LC/MS System |
| --- | --- |
| Gas temperature | 120°C |
| Gas flow | 12 L/min |
| Nebulizer | 50 psi |
| Sheath gas temperature | 400°C |
| Sheath gas flow | 12 L/min |
| Capillary voltage | 3500 V |
| Nozzle voltage | 0 V |
| Cycle time | 500 ms |
| Total MRMs | 10 |
| Mass transitions  (precursor to product) | *O*^6^-MeG; *m*/*z* 166.1 > 149.0  *N*^3^-MeA; *m*/*z* 150.0 > 123.0 |

【Stability of DNA adducts (*O*^6^-MeG, *N*^3^-MeA) in the presence of the test compounds 】

*O*^6^-MeG (5 mM) and *N*^3^-MeA (5 mM) were dissolved in methanol. The each solutions were diluted with 5% aqueous formic acid solution to make 10 μM concentrations.

*O*^6^-MeG (3.6 μM in 5% aqueous formic acid solution), *N*^3^-MeA (3.6 μM in 5% aqueous formic acid solution), a test compound (3.6 μM in DMSO) were prepared. Each solution (100 μL; final concentration 1.2 μM) were mixed and incubated for 16 h at 37°C. DMSO (100 μL) was used instead of the compound solution as a control. The solutions were analyzed LC-MS/MS (Table S10 and S11).

**Table S11** Stability of *O^6^*-MeG and *N^3^*-MeA in the presence of the compounds

|  | *O* ^6^-MeG (%) | *N* ^3^-MeA (%) |
| --- | --- | --- |
| Control | 100.0 | 100.0 |
| + Licoricidin | 98.7 | 98.7 |
| + Isoliquiritigenin | 97.8 | 98.9 |
| + 4’,6,7-Trihydroxyisoflavone | 98.1 | 97.8 |
| + Glycyrrhetinic acid | 98.8 | 96.9 |
| + Ethidium bromide | 97.5 | 95.4 |
| + Hoechst 33258 | 97.0 | 94.2 |

【Stability of DNA adducts (*O*^6^-MeG, *N*^3^-MeA) under acid hydrolysis conditions】

*O*^6^-MeG (5 mM) and *N*^3^-MeA (5 mM) were dissolved in methanol. The each solutions were diluted with 5% aqueous formic acid solution to prepare 1.0 μM concentrations.

Each solution (50 μL, final concentration 0.5 μM) were mixed and heated for 1 h at 85°C. The solutions were analyzed LC-MS/MS (Table S11 and S13).

**Table S12**  Stability of *O^6^*-MeG and *N^3^*-MeA under acid hydrolysis conditions

|  | *O* ^6^-MeG (%) | *N* ^3^-MeA (%) |
| --- | --- | --- |
| Control | 100.0 | 100.0 |
| Acid hydrolysis conditions | 97.0 | 99.3 |

**Table S13** Quantification of *O^6^*-MeG and *N^3^*-MeA in the reaction mixture of MNU and licoricidin

| Concentration | *O*^6^-MeG | | *N*^3^-MeA | |
| --- | --- | --- | --- | --- |
| (mM) | Adducts 10^5^ ± SE | Percentage (%) | Adducts 10^5^ ± SE | Percentage (%) |
| 0 | 5691 ± 185 | 100.0 | 372 ± 18 | 100.0 |
| 0.2 | 5137 ± 131 | 90.3 | 317 ± 10 | 85.4 |
| 1.0 | 4870 ± 199 | 85.6 | 268 ± 24 | 72.0 |
| 2.0 | 4643 ± 194 | 81.7 | 253 ± 7 | 68.1 |
| 3.0 | 4896 ± 121 | 86.1 | 243 ± 12 | 65.5 |
| 4.0 | 4502 ± 217 | 79.2 | 210 ± 12 | 56.6 |

**Table S14** Quantification of *O^6^*-MeG and *N^3^*-MeA in the reaction mixture of MNU and isoliquiritigenin

| Concentration | *O*^6^-MeG | | *N*^3^-MeA | |
| --- | --- | --- | --- | --- |
| (mM) | Adducts 10^5^ ± SE | Percentage (%) | Adducts 10^5^ ± SE | Percentage (%) |
| 0 | 6615 ± 359 | 100.0 | 459 ± 12 | 100.0 |
| 0.2 | 6084 ± 102 | 93.5 | 417 ± 9 | 90.9 |
| 1.0 | 4518 ± 437 | 69.2 | 309 ± 13 | 67.3 |
| 2.0 | 4058 ± 50 | 62.4 | 268 ± 2 | 58.5 |
| 4.0 | 3497 ± 162 | 53.7 | 217 ± 3 | 47.2 |
| 8.0 | 2579 ± 130 | 39.6 | 159 ± 10 | 34.7 |

**Table S15** Quantification of *O^6^*-MeG and *N^3^*-MeA in the reaction mixture of MNU and 4’,6,7-trihydroxyisoflavone

| Concentration | *O*^6^-MeG | | *N*^3^-MeA | |
| --- | --- | --- | --- | --- |
| (mM) | Adducts 10^5^ ± SE | Percentage (%) | Adducts 10^5^ ± SE | Percentage (%) |
| 0 | 6163 ± 125 | 100.0 | 539 ± 23 | 100.0 |
| 0.2 | 4954 ± 167 | 80.4 | 400 ± 13 | 74.3 |
| 1.0 | 3831 ± 99 | 62.2 | 272 ± 13 | 50.6 |
| 2.0 | 3249 ± 33 | 52.7 | 199 ± 2 | 36.9 |
| 4.0 | 3416 ± 114 | 55.4 | 188 ± 8 | 34.9 |
| 8.0 | 3343 ± 114 | 54.2 | 176 ± 6 | 32.8 |

**Table S16** Quantification of *O^6^*-MeG and *N^3^*-MeA in the reaction mixture of MNU and glycyrrhetinic acid

| Concentration | *O*^6^-MeG | | *N*^3^-MeA | |
| --- | --- | --- | --- | --- |
| (mM) | Adducts 10^5^ ± SE | Percentage (%) | Adducts 10^5^ ± SE | Percentage (%) |
| 0 | 3918 ± 95 | 100.0 | 341 ± 11 | 100.0 |
| 0.2 | 3560 ± 25 | 91.6 | 280 ± 6 | 81.9 |
| 1.0 | 3348 ± 41 | 85.5 | 293 ± 8 | 85.8 |
| 2.0 | 3237 ± 106 | 82.6 | 303 ± 10 | 88.8 |
| 4.0 | 3774 ± 66 | 96.3 | 300 ± 18 | 87.9 |
| 8.0 | 3491 ± 111 | 89.1 | 302 ± 11 | 88.6 |

**Table S17** Quantification of *O^6^*-MeG and *N^3^*-MeA in the reaction mixture of MNU and

ethidium bromide

| Concentration | *O*^6^-MeG | | *N*^3^-MeA | |
| --- | --- | --- | --- | --- |
| (mM) | Adducts 10^5^ ± SE | Percentage (%) | Adducts 10^5^ ± SE | Percentage (%) |
| 0 | 4850 ± 87 | 100.0 | 377 ± 12 | 100.0 |
| 0.02 | 7795 ± 362 | 160.7 | 292 ± 3 | 77.5 |
| 0.04 | 8927 ± 560 | 184.1 | 221 ± 12 | 58.7 |
| 0.2 | 4740 ± 117 | 97.7 | 55 ± 5 | 14.6 |
| 1.0 | 1530 ± 26 | 31.5 | ― | ― |
| 2.0 | 502 ± 96 | 10.3 | ― | ― |

**Table S18** Quantification of *O^6^*-MeG and *N^3^*-MeA in the reaction mixture of MNU and Hoechst 33258

| Concentration | *O*^6^-MeG | | *N*^3^-MeA | |
| --- | --- | --- | --- | --- |
| (mM) | Adducts 10^5^ ± SE | Percentage (%) | Adducts 10^5^ ± SE | Percentage (%) |
| 0 | 1291 ± 180 | 100.0 | 199 ± 22 | 100.0 |
| 0.2 | 1320 ± 255 | 102.3 | 211 ± 19 | 105.9 |
| 1.0 | 1192 ± 105 | 92.4 | 158 ± 10 | 79.2 |
| 2.0 | 821 ± 87 | 63.6 | 81 ± 4 | 40.6 |
| 4.0 | 507 ± 159 | 39.3 | 54 ± 13 | 27.0 |
| 8.0 | 251 ± 42 | 19.5 | 25 ± 7 | 12.4 |

**Table S19** Decrease of MNU

| Time (min) | MNU alone |
| --- | --- |
| 0 | 175558.3 ± 7896.2 |
| 20 | 107837.7 ± 4537.8 |
| 40 | 63616.0 ± 5772.3 |
| 60 | 37865.7 ± 3515.9 |
| 80 | 20973.3 ± 3777.3 |
| 100 | 12135.3 ± 2244.5 |
| 120 | 7136.3 ± 686.2 |

**Table S20** Decrease of MNU in the presence of licoricidin or isoliquiritigenin

| Time (min) | Licoricidin | Isoliquiritigenin |
| --- | --- | --- |
| 0 | 186675.3 ± 10774.6 | 173462.3 ± 1596.9 |
| 20 | 103709.0 ± 6308.3 | 96584.0 ± 1519.8 |
| 40 | 62003.3 ± 11960.2 | 56391.0 ± 1920.1 |
| 60 | 36875.3 ± 3694.6 | 32932.3 ± 2396.6 |
| 80 | 20709.7 ± 3682.1 | 18763.3 ± 1051.9 |
| 100 | 11387.0 ± 2546.2 | 10113.3 ± 873.8 |
| 120 | 6369.0 ± 1518.6 | 5630.0 ± 67.9 |

**Table S21** Decrease of MNU in the presence of 4’,6,7-trihydroxyisoflavone or glycyrrhetinic acid

| Time (min) | 4’,6,7-Trihydroxyisoflavone | Glycyrrhetinic acid |
| --- | --- | --- |
| 0 | 157257.0 ± 13302.5 | 145948.0 ± 10659.0 |
| 20 | 68648.3 ± 9372.2 | 89414.3 ± 6621.8 |
| 40 | 31237.0 ± 6760.7 | 50378.7 ± 2558.8 |
| 60 | 13780.7 ± 3810.3 | 28778.3 ± 5580.2 |
| 80 | 5751.0 ± 1730.6 | 20998.3 ± 1934.9 |
| 100 | ND | 14948.0 ± 567.4 |
| 120 | ND | 8706.0 ± 2970.2 |

**Table S22** Decrease of MNU in the presence of ethidium bromide or Hoechst 33258

| Time (min) | Ethidium bromide | Hoechst 33258 |
| --- | --- | --- |
| 0 | 172754.7 ± 7584.7 | 173418.7 ± 3655.5 |
| 20 | 69844.0 ± 3882.6 | 103208.0 ± 13574.7 |
| 40 | 31257.7 ± 4255.3 | 58691.0 ± 9930.3 |
| 60 | 11832.0 ± 1882.6 | 32956.7 ± 6688.4 |
| 80 | 4863.3 ± 813.7 | 18418.0 ± 2877.1 |
| 100 | ND | 9699.7 ± 1636.8 |
| 120 | ND | 5426.7 ± 853.3 |


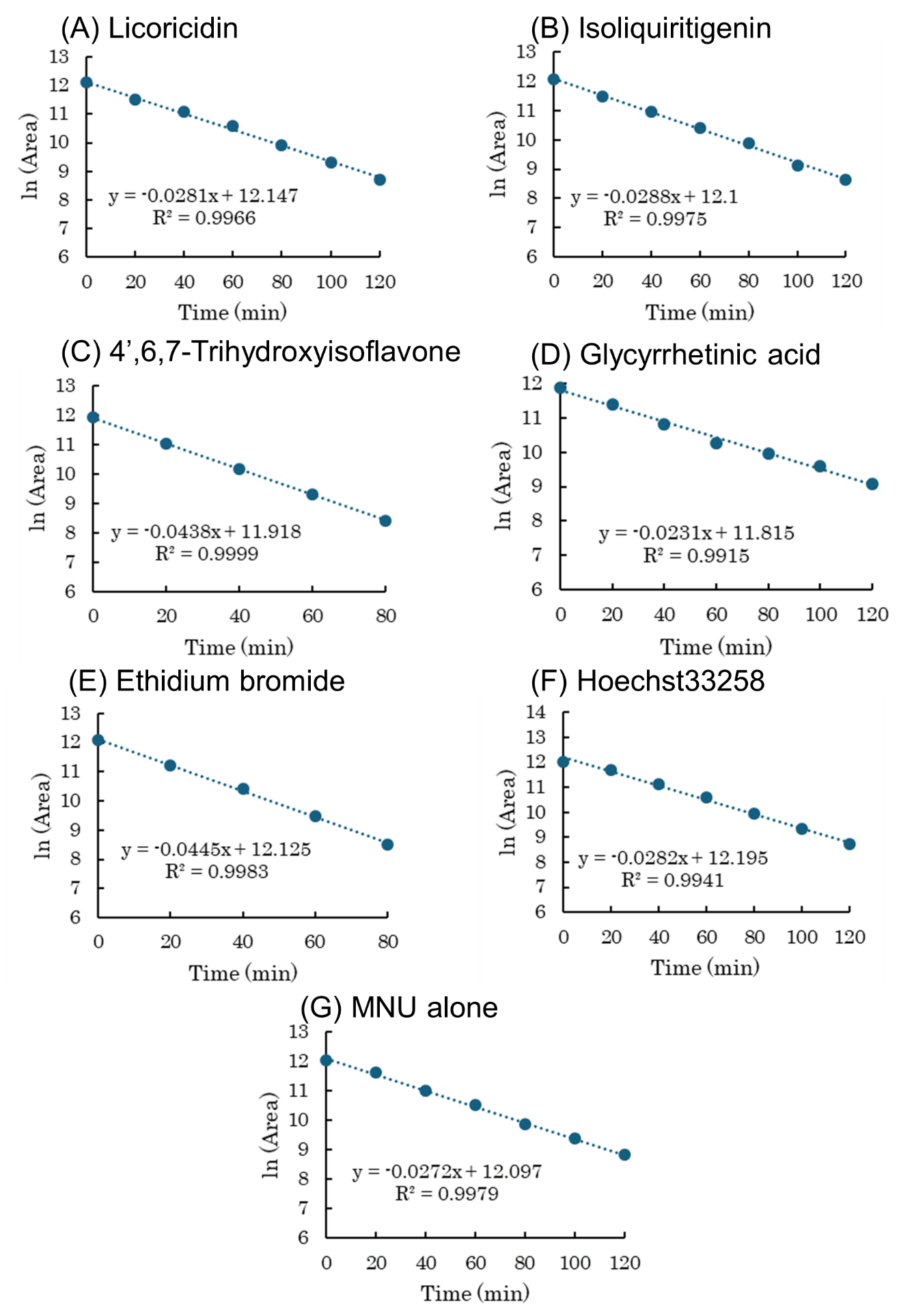


**Figure S6** Plots of ln (MNU) versus time

**Table S23** Quantification of the remaining licoricidin in the presence or absence of MNU

| Time | MNU (－) | | MNU (+) | |
| --- | --- | --- | --- | --- |
| (h) | Area | Remaining (%) | Area | Remaining (%) |
| 0 | 138423 ± 4356 | 100.0 ± 0.0 | 149243 ± 15732 | 100.0 ± 0.0 |
| 1 | 138522 ± 5093 | 100.1 ± 0.7 | 147852 ± 11785 | 99.8 ± 2.7 |
| 2 | 136177 ± 3684 | 98.4 ± 0.6 | 146932 ± 12416 | 99.1 ± 2.2 |
| 3 | 134811 ± 6326 | 97.4 ± 1.7 | 147511 ± 14674 | 99.0 ± 0.9 |
| 4 | 133674 ± 4154 | 96.6 ± 0.2 | 148183 ± 15560 | 99.3 ± 0.4 |
| 5 | 131523 ± 3874 | 95.0 ± 1.4 | 142661 ± 12740 | 96.0 ± 1.7 |
| 6 | 129923 ± 3697 | 93.6 ± 0.5 | 143843 ± 12855 | 96.8 ± 1.4 |
| 24 | 126511 ± 8645 | 91.4 ± 3.4 | 136447 ± 13947 | 91.5 ± 2.0 |

**Table S24** Quantification of the remaining isoliquiritigenin in the presence or absence and of MNU

| Time | MNU (－) | | MNU (+) | |
| --- | --- | --- | --- | --- |
| (h) | Area | Remaining (%) | Area | Remaining (%) |
| 0 | 222886 ± 18519 | 100.0 ± 0.0 | 222879 ± 18514 | 100.0 ± 0.0 |
| 1 | 220540 ± 33374 | 97.3 ± 6.7 | 230822 ± 35331 | 101.7 ± 8.3 |
| 2 | 231212 ± 27480 | 102.9 ± 3.7 | 221341 ± 34088 | 97.6 ± 8.9 |
| 3 | 228409 ± 32091 | 101.0 ± 5.8 | 249281 ± 21748 | 102.5 ± 3.8 |
| 4 | 220680 ± 37711 | 97.3 ± 9.1 | 236406 ± 37017 | 104.3 ± 10.6 |
| 5 | 225881 ± 28550 | 100.2 ± 4.4 | 226162 ± 37749 | 99.3 ± 8.9 |
| 6 | 216309 ± 35156 | 95.1 ± 7.8 | 234905 ± 37088 | 103.4 ± 9.3 |
| 24 | 222090 ±32769 | 98.2 ± 6.4 | 216094 ± 29762 | 95.6 ± 5.9 |

**Table S25** Quantification of the remaining 4’,6,7-trihydroxyisoflavone in the presence or absence of MNU

| Time | MNU (－) | | MNU (+) | |
| --- | --- | --- | --- | --- |
| (h) | Area | Remaining (%) | Area | Remaining (%) |
| 0 | 121580 ± 4356 | 100.0 ± 0.0 | 139239 ± 18514 | 100.0 ± 0.0 |
| 1 | 117414 ± 5093 | 97.0 ± 2.9 | 126311± 35331 | 91.0 ± 1.9 |
| 2 | 121024 ± 3684 | 99.8 ± 2.3 | 115991 ± 34088 | 84.1 ± 5.0 |
| 3 | 121599 ± 6326 | 100.1 ± 0.7 | 116729 ± 21748 | 84.3 ± 2.8 |
| 4 | 120114 ± 4154 | 99.0 ± 1.8 | 115947 ± 37017 | 83.5 ± 1.6 |
| 5 | 121445 ± 3874 | 100.1 ± 1.4 | 118077 ± 37749 | 85.1 ± 2.2 |
| 6 | 122007 ± 3697 | 100.5 ± 1.8 | 114530± 37088 | 82.5 ± 1.7 |
| 24 | 116421 ± 8645 | 96.0 ± 0.6 | 107623 ± 29762 | 77.6 ± 2.3 |

**Table S26** Quantification of the remaining glycyrrhetinic acid in the presence or absence of MNU

| Time | MNU (－) | | MNU (+) | |
| --- | --- | --- | --- | --- |
| (h) | Area | Remaining (%) | Area | Remaining (%) |
| 0 | 9213188 ± 291900 | 100.0 ± 0.0 | 9004170 ± 200746 | 100.0 ± 0.0 |
| 1 | 8572393 ± 130607 | 93.2 ± 1.6 | 9142155 ± 119624 | 101.7 ± 2.2 |
| 2 | 9160857 ± 212929 | 99.6 ± 2.9 | 8820081 ± 166809 | 98.0 ± 0.3 |
| 3 | 9337042 ± 356112 | 101.4 ± 2.4 | 9371942 ± 64427 | 104.1 ± 1.6 |
| 4 | 8949650 ± 395606 | 97.1 ± 2.1 | 9246317 ± 116036 | 102.7 ± 2.8 |
| 5 | 9216296 ± 296150 | 100.2 ± 3.3 | 9077629 ± 82486 | 100.8 ± 2.4 |
| 6 | 9249813 ± 393978 | 100.4 ± 3.0 | 9228687 ± 28111 | 102.6 ± 2.3 |
| 24 | 9398528 ± 255443 | 102.2 ± 2.9 | 9173247 ± 184407 | 101.9 ± 1.9 |

**References**

1. Hu C, Lin B, Chao M. Quantitative determination of urinary *N*^3^-methyladenine by isotope-dilution LC–MS/MS with automated solid-phase extraction. Int J Mass Spectrom. 2011;304:68–73.
2. Zhang F, Bartels MJ, Pottenger LH, Gollapudi BB, Schisler MR. Simultaneous quantitation of 7-methyl- and *O*^6^-methylguanine adducts in DNA by liquid chromatography–positive electrospray tandem mass spectrometry. J Chromatogr B. 2006; 833:141–8.
